# Tuberculous Spondylodiscitis with Sacroiliac Joint Involvement

**DOI:** 10.1590/0037-8682-0363-2025

**Published:** 2026-02-09

**Authors:** Sadullah Şimşek, Mehmet Salih Karaca, Tarık Sağlam

**Affiliations:** 1 Gazi Yasargil Training and Research Hospital, Department of Radiology, Diyarbakır, Türkiye.

**FIGURE 1:**
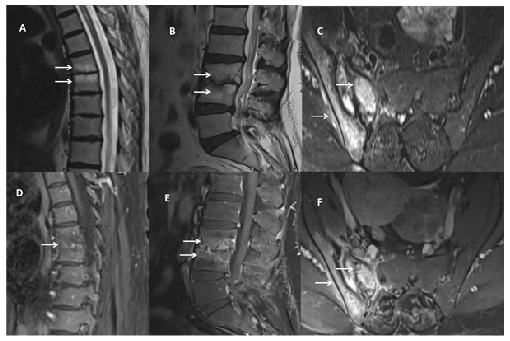
Magnetic resonance imaging of the thoracic and lumbar spine and right sacroiliac joint. T2-weighted images **(A-C)** demonstrate areas of high signal intensity (arrows), while postcontrast images **(D-F)** show corresponding contrast enhancement **(arrows)**.

**FIGURE 2:**
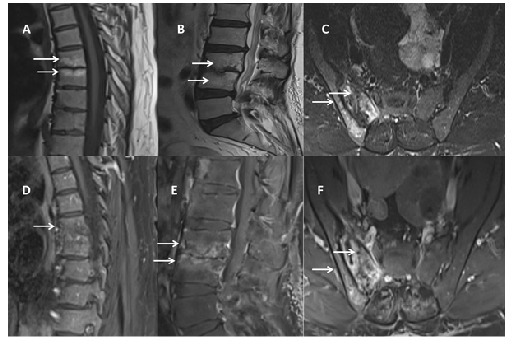
Follow-up magnetic resonance imaging after 6 months of antituberculosis treatment reveals decreased signal intensity on T2-weighted images **(A-C, arrows)** and reduced contrast enhancement on postcontrast images **(D-F, arrows)**.

A 50-year-old woman presented with a 1 -year history of progressively worsening low back and hip pain, accompanied by a 10 kg weight loss. Laboratory evaluation revealed elevated inflammatory markers (erythrocyte sedimentation rate: 27 mm/h; C-reactive protein: 22 mg/L). Contrast-enhanced magnetic resonance imaging (MRI) revealed endplate irregularities, edema, disc space narrowing, and vertebral enhancement at T6-7, consistent with spondylitis. At L3-L4, MRI showed endplate destruction, edema, and intervertebral disc damage with marked enhancement, indicative of spondylodiscitis. T2-weighted images revealed hyperintense signals in the right sacroiliac joint, with postcontrast enhancement of the joint, adjacent bone, and surrounding soft tissues, consistent with sacroiliitis (Figure 1). A Quantiferon test performed for suspected tuberculosis was positive (TB2 antigen: 0.98). The patient was treated with a 6-month antituberculosis regimen, leading to clinical improvement and marked radiologic regression on follow-up MRI (Figure 2).

Bone and joint tuberculosis constitutes a small proportion of all tuberculosis cases but can cause substantial morbidity. Spinal tuberculosis is the most common form, with an incidence of 2.4-7.2 per 100,000 in developed countries^1^. Sacroiliac joint involvement is uncommon, accounting for 3-9.7% of musculoskeletal tuberculosis^2^. Coexisting sacroiliac and spinal involvement complicates diagnosis.
